# Peroxiredoxin 6 Knockout Mice Demonstrate Anxiety Behavior and Attenuated Contextual Fear Memory after Receiving Acute Immobilization Stress

**DOI:** 10.3390/antiox10091416

**Published:** 2021-09-04

**Authors:** Sarayut Phasuk, Peeraporn Varinthra, Andaman Nitjapol, Korakod Bandasak, Ingrid Y. Liu

**Affiliations:** 1 Institute of Medical Sciences, Tzu Chi University, Hualien 970, Taiwan; 103353118@gms.tcu.edu.tw (S.P.); vpeeraporn@gms.tcu.edu.tw (P.V.); 2 Department of Physiology, Faculty of Medicine Siriraj Hospital, Mahidol University, Bangkok 10700, Thailand; 3 Faculty of Medical Science, Naresuan University, Phitsanulok 65000, Thailand; andamann62@nu.ac.th (A.N.); korakodb62@nu.ac.th (K.B.)

**Keywords:** peroxiredoxin 6, acute immobilization stress, stress, fear memory, glucocorticoid

## Abstract

Stress can elicit glucocorticoid release to promote coping mechanisms and influence learning and memory performance. Individual memory performance varies in response to stress, and the underlying mechanism is not clear yet. Peroxiredoxin 6 (PRDX6) is a multifunctional enzyme participating in both physiological and pathological conditions. Several studies have demonstrated the correlation between PRDX6 expression level and stress-related disorders. Our recent finding indicates that lack of the *Prdx6* gene leads to enhanced fear memory. However, it is unknown whether PRDX6 is involved in changes in anxiety response and memory performance upon stress. The present study reveals that hippocampal PRDX6 level is downregulated 30 min after acute immobilization stress (AIS) and trace fear conditioning (TFC). In human retinal pigment epithelium (ARPE-19) cells, the PRDX6 expression level decreases after being treated with stress hormone corticosterone. Lack of PRDX6 caused elevated basal H_2_O_2_ levels in the hippocampus, basolateral amygdala, and medial prefrontal cortex, brain regions involved in anxiety response and fear memory formation. Additionally, this H_2_O_2_ level was still high in the medial prefrontal cortex of the knockout mice under AIS. Anxiety behavior of *Prdx6*^−/−^ mice was enhanced after immobilization for 30 min. After exposure to AIS before a contextual test, *Prdx6^−/−^* mice displayed a contextual fear memory deficit. Our results showed that the memory performance of *Prdx6^−/−^* mice was impaired when responding to AIS, accompanied by dysregulated H_2_O_2_ levels. The present study helps better understand the function of PRDX6 in memory performance after acute stress.

## 1. Introduction

Acute stress can either facilitate or impair cognitive performance depending on a combination of factors, such as timing related to stress and the types of cognitive function [1,2]. The hypothalamic-pituitary–adrenal (HPA) axis is activated to release stress hormone glucocorticoid (GC) into the bloodstream and subsequently to liberate stored energy required for stress-coping mechanisms [3,4]. Amario A. et al. reported that elevated glucose, cortisol, and prolactin in the bloodstream are markers in responding to acute immobilization stress (AIS) [5]. Stress-induced elevated energy metabolism leads to the generation of reactive oxygen species (ROS) [6]. The hippocampus, a critical brain region involved in learning and memory, is sensitive to stress hormones and oxidative stress [7,8,9]. Previous studies indicate that both glucocorticoid receptors (GRs) and mineralocorticoid receptors (MRs) are highly expressed in the hippocampus [10,11]. These studies suggest that the hippocampus is a critical brain region responsible for stress response, and GC is implicated in memory performance [12,13]. Under stressful conditions, various factors influence the effects of stress on an individual’s memory performance [14]. Thus, finding the key molecules responsible for the complex relationship between acute stress and memory formation will help better understand the underlying mechanism.

Peroxiredoxin 6 (PRDX6), a 25-kDa protein, is the sixth member of the peroxiredoxin family [15]. It is the only mammalian peroxiredoxin that contains one active cysteine residue and does not require thioredoxin in its catalytic cycle [15]. PRDX6 exerts glutathione peroxidase (GPx) and acidic calcium-independent PLA2 (aiPLA2) activities [16,17,18], which define its potential role in oxidative regulation and membrane lipid turnover [16,19,20]. PRDX6 is expressed in the brain, including the hippocampus [21], amygdala, and prefrontal cortex [22]. The *PRDX6* gene contains glucocorticoid responsive elements (GRE) on its promoter region. It can be upregulated by dexamethasone (Dex), a selective GR agonist, in lung epithelium cells [23,24]. Previous studies also showed that immunophilin FK506-binding protein 52 (FKBP52), a chaperone protein promoting nuclear translocation of GRs, positively correlates with PRDX6 level. Deletion of the *Fkbp4* gene results in reduced PRDX6 levels [25,26]. PRDX6 is involved in various forms of stress-related disorders [21,27,28]. Our previous studies have identified the PRDX6 function in modulating hippocampal synaptic plasticity and memory formation [29,30]. Thus, it is worth investigating whether PRDX6 is involved in stress-related anxiety response and memory performance.

The present study demonstrates that PRDX6 expression level was downregulated in response to corticosterone treatment in vitro, and after stressful training in vivo. After receiving AIS, the H_2_O_2_ level is decreased in the *Prdx6^−/−^* mice brains. Additionally, AIS induces anxiety in *Prdx6^−/−^* mice while attenuating their excessive fear of memory retrieval.

## 2. Materials and Methods

### 2.1. Animals

Male mice used in this study were 8–12-weeks old. Mice with a targeted deletion of the *Prdx6* gene were purchased from the Jackson Laboratory (#005974 B6.129-Prdx6 tm1pgn/pgn, Bar Harbor, NE, USA) and maintained at Tzu Chi University for more than 10 generations. The generation of the *Prdx6* mice was described in the previous report [31]. One heterozygous (*Prdx6^+/−^*) male and two heterozygous (*Prdx6^+/−^*) female knockout mice were mated to generate homozygous wild–type (*Prdx6^+/+^*) littermates and knockout (*Prdx6^−/−^*) mice. Genotyping was performed as described in the previous report to confirm the genotypes of the mice before every behavioral test [29]. All mice were kept in normal laboratory conditions and had free access to food and water under a 12 h light/dark cycle. All experiments in this study were conducted following the ethical guidelines of the Taiwan Ministry of Science and Technology (MOST) (Taipei, Taiwan) and approved by the Institutional Animal Care and Use Committee of Tzu Chi University, Hualian, Taiwan (approval #104099-A, 24 January 2018). The ethical treatment of animals followed the guidelines provided by Taiwan MOST.

### 2.2. Behavioral Tests

#### 2.2.1. Acute Immobilization Stress (AIS)

To restrain the mice, we kept them in 50 mL plastic conical tubes with breathing holes for 30 min. A non-transparent paper box was used to cover them for mimicking the dark phase [13]. After 30 min of immobilization, stressed mice were returned to their home cage and rested for 20 min. After the behavioral test, mice were sacrificed immediately for hippocampal tissue collection.

#### 2.2.2. Trace Fear Conditioning

We performed trace fear conditioning as described in our previous study with minor modification [21]. Mice were habituated to the conditioning apparatus (17 cm (W) × 17 cm (L) × 25 cm (H)) for 15 min three consecutive days. On the next day, three pairs of tone (CS) and electric foot shock (US) were used to train the mice. Each pair consisted of 20 s tone (6000 Hz, 85 dB) and 1 s electric foot shock (1 mA) with a 10 s training interval. Twenty-four hours later, mice were re-exposed to the conditioned chamber for 6 min to test their contextual fear memory. A video camera recorded the testing procedure, and the freezing percentage was analyzed by tracking software (EthoVision XT 15, Noldus Information Technology, Leesburg, VA, USA).

#### 2.2.3. Open Field Test

To test mice’s locomotor function and anxiety response, they were placed into an open field chamber (50 cm × 50 cm × 50 cm) and allowed to explore the chamber for 10 min freely [21]. A top-view camera was used to record the traveling distance and moving speed. The chamber was divided into three zones: outer, inner, and center. The time spent in each zone was calculated using tracking software (EthoVision XT 15, Noldus Information Technology, Leesburg, VA, USA).

#### 2.2.4. Elevated plus Maze Test

We used an elevated plus-maze to evaluate the fear of height. Mice were placed in the center of the 60 cm high maze [21] and allowed to freely explore the maze for 10 min. Their motion was recorded by a top-view camera and analyzed by tracking software (EthoVision XT 15, Noldus Information Technology, Leesburg, VA, USA) to obtain time spent in closed arms and open arms.

### 2.3. Cell Culture

ARPE-19 was kindly provided by Rong-Kung Tsai at the Institute of Medical Sciences, Tzu Chi University, Taiwan. The cells were initially purchased from the Bioresource Collection and Research Center (BCRC, Hsinchu, Taiwan). In this study, the cells were cultured in a medium comprising 10% fetal bovine serum (FBS) in Dulbecco’s Modified Eagle Medium: nutrient mixture F-12 (DMEM/F12), 100 U/mL penicillin, and 100 ug/mL streptomycin. The cells were maintained in a 37 °C humidified incubator with 5% CO_2_ atmosphere. Cells were seeded in 96-well plates for 24 h to achieve 80% confluence. A 0.01% DMSO or varying doses of corticosterone (1, 10 and 100 nM) was added into the medium for 1 h. Thiazolyl Blue Tetrazolium Blue (MTT) was then conducted to measure cell viability. Briefly, 10 µL of MTT solution (5 mg/mL) in phosphate-buffered saline (PBS) was added to each well and incubated for 3 h at 37 °C. The supernatant was removed and replaced by 100 µL DMSO. A 570 nm wavelength was used to obtain the intensity of MTT under a microplate reader (Thermo Scientific Multiskan Spectrum, Waltham, MA, USA).

### 2.4. Immunocytochemistry and Image Analysis

For immunocytochemistry, ARPE-19 cells (5 × 10^3^) were seeded in 24-well plates containing 10 mm coverslips and maintained in an incubator at 37 °C overnight. After that, GC was added to the cells for 1 h, then cells were washed three times with 1× PBS and fixed with 4% paraformaldehyde (PFA) for 30 min at room temperature. Fixed cells were washed three times with a washing buffer (1× PBS containing 0.3% Triton X-100), then added with a blocking buffer (1 mg/mL BSA containing 0.3% Triton X-100) for 1 h at room temperature on a shaker. Next, cells were incubated with 200 µL of monoclonal mouse anti-PRDX6 antibody (1:200, Bethyl Laboratories, Inc, Montgomery, TX, USA) overnight at 4 °C. Next, cells were washed three times with a washing buffer for 10 min/time. The fixed cells were then incubated in a secondary antibody (Alexa 488-conjugated goat anti-rabbit IgG (1:200, ThermoFisher Scientific, Waltham, MA, USA)) for 1 h followed by washing with the washing buffer. The stained cells were counterstained with DAPI (1:10,000) for 5 min. The images were observed under a fluorescent microscope (Nikon model# ECLIPSE Ni-E, Tokyo, Japan). The percentage of labeled cells (450 μm × 450 μm) was quantified using ImageJ software version 15.2a (download from National Institutes of Health, Bethesda, MD, USA).

### 2.5. Western Blot (WB) Analysis

WB analysis procedure was the same as described in our previous study [29]. The hippocampal tissues were lysed in 1X radioimmunoprecipitation assay (RIPA) buffer (Merck Millipore, Burlington, MA, USA) containing phosphatase and protease inhibitors and kept on ice for 30 min. The lysates were then centrifuged at 13,000 rpm for 15 min at 4 °C. Protein samples (30 μg) were collected in 1x sample buffer (SB) with 10% reducing agent (RA) and separated by 10% SDS-PAGE. Proteins were then transferred to a PVDF membrane (0.22 μm pore size). After that, the members were washed three times with 1X PBS containing 0.1% Tween-20. We probed the proteins of interest with corresponding primary antibody: monoclonal mouse anti-PRDX6 (1:2000, Bethyl laboratories, Inc, USA) and secondary antibodies: HRP-conjugated goat anti-mouse antibody (1:10,000, cell signaling technology, Danvers, MA, USA). The enhanced chemiluminescence reagents (Western Lightning^®^ Plus-ECL, PerkinElmer, MA, USA) were used before detecting blots under the UVP Biospectrum 810 imaging system to visualize proteins at a specific molecular weight. The intensities of protein bands were quantified using ImageJ software version 15.2a (National Institutes of Health, Bethesda, MD, USA) to measure protein expression levels.

### 2.6. Intracellular ROS Accumulation Measurement

After AIS, the brain tissues were fixed with 4% PFA overnight at room temperature before being submerged in 30% sucrose at 4 °C until sinkage. The cryopreserved brains were cut coronally by cryotome with 20 μm thickness. The sections were selected based on brain coordinates: at bregma 1.98 to 2.34 mm for medial prefrontal cortex (mPFC) and −1.28 to −2.92 mm for hippocampus and basolateral amygdala (BLA). Selected sections were stained with 1 μM of dihydroethidium (DHE) for 5 min at room temperature followed by three times of washing in 1× PBS, 10 min each and cover-slipped. To measure the level of intracellular H_2_O_2_, the stained sections were imaged under the confocal microscope (Nikon model#C2^+^, Japan). For calculating the fluorescent intensity in each brain area, 3 fields (200 × 200 μm) from 3 sections per mouse were quantified using ImageJ software (National Institutes of Health, Bethesda, MD, USA).

### 2.7. Statistical Analysis

The SPSS (version 25, IBM Corporation, Armonk, NY, USA) was used for statistical analysis, and the graphs were made using GraphPad Prism version 8.0 (San Diego, CA, USA). All data are plotted as mean ± standard error of the mean (mean ± SEM) with 95% confidence interval as statistically significant (*p* < 0.05). Student’s *t*-tests were used to compare the data of two independent groups. For multiple comparisons, we performed a one-way ANOVA followed by a Bonferroni post hoc test. The mixed-design repeated measures ANOVA was used to analyze the data of related dependent groups. The sample sizes for each experiment are shown in figure legends.

## 3. Results

### 3.1. PRDX6 Expression Level Was Decreased in ARPE-19 Cells Treated with Stress Hormone Glucocorticoid

**Figure 1:**
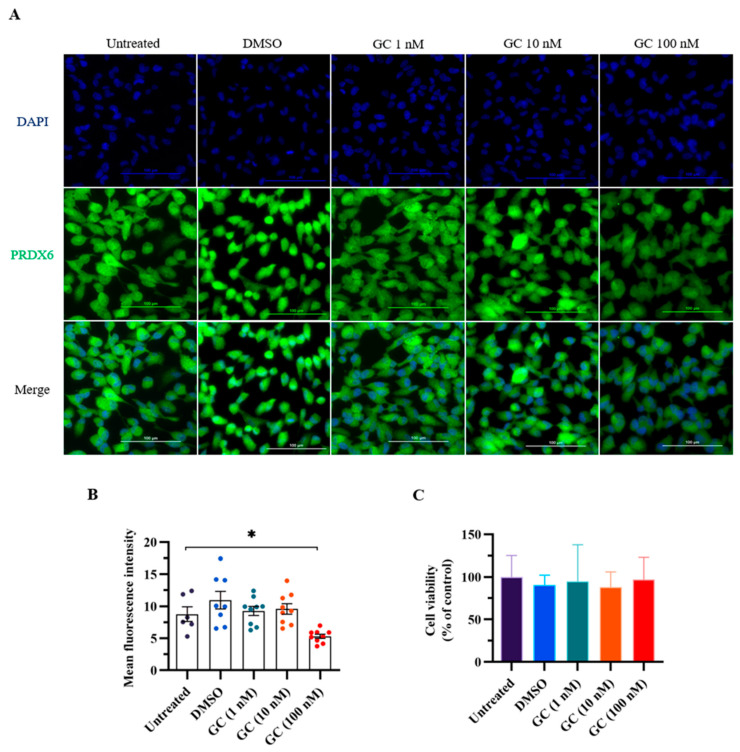
PRDX6 levels in response to 1 h of glucocorticoid (GC) treatment at 1, 20, and 100 nM doses. (**A**) Immunofluorescent images with 400× magnification of PRDX6 expressed in ARPE-19 cells after administration of GC. (**B**) Quantification of PRDX6 expression levels after GC treatments (*n* = 6–9 per group, one-way ANOVA followed by Bonferroni’s post hoc test). (**C**) Viability of ARPE-19 cells treated with GC. All data represent the mean ± the SEM. * *p* < 0.05. GC, glucocorticoid; PRDX6, peroxiredoxin 6; DAPI, 4′,6-diamidino-2-phenylindole.

We first conducted an in vitro experiment to evaluate the effect of stress hormone glucocorticoid on the expression level of PRDX6. We added GC to ARPE-19 cells, which express PRDX6 protein and have steroid receptors [32,33]. We added glucocorticoids of 1, 20, and 100 nM to the cells to mimic stress stimulation in vitro. Interestingly, PRDX6 expression level was significantly reduced under 100 nM GC treatment according to the immunostaining results (Figure 1A,B) (*F*_4,25_ = 4.506; *p* = 0.008). To understand whether GC treatment causes cytotoxicity, we performed MTT assay, and found that cell viability was similar among groups (Figure 1C) (*F*_4,25_ = 0.191; *p* = 0.941).

### 3.2. PRDX6 Expression Level Was Downregulated in Response to AIS and TFC

**Figure 2:**
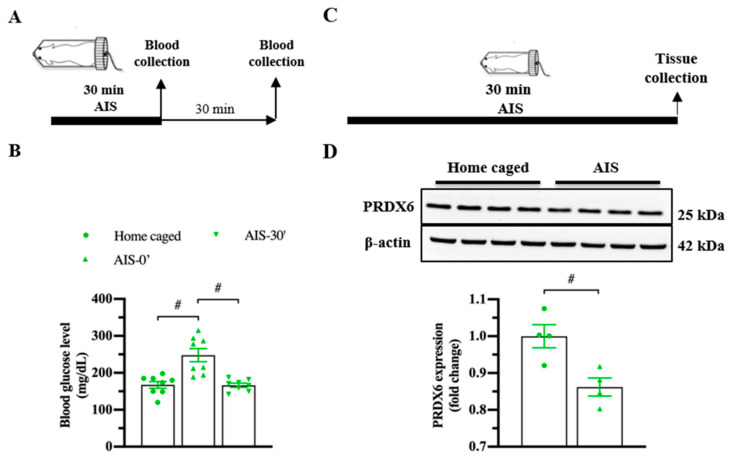
PRDX6 expression and blood glucose levels in response to acute immobilization stress (AIS). (**A**) The procedure of AIS treatment and blood glucose measurement. (**B**) Blood glucose level (mg/dL) of home caged, immediately and 30 min after completing AIS. Blood was collected from the tail vein (*n* = 8 per group, one-way ANOVA followed by Bonferroni’s post hoc test). (**C**) Tissue samples were collected from the hippocampus immediately after the completion of immobilization. (**D**) Immunoblots of PRDX6 and β-actin expression levels in the hippocampus (*n* = 4 per group). Quantification data for PRDX6 expression levels (Student’s *t*-test) after receiving 30 min of AIS. All data represent the mean ± the SEM. # *p* < 0.05. PRDX6, peroxiredoxin 6; AIS, acute immobilization stress.

To investigate whether PRDX6 responds to acute stress, we measured blood glucose level (Figure 2A; upper panel) and expression levels of PRDX6 after AIS (Figure 2C; upper panel). By 30 min of immobilization, we observed a significant alteration of blood glucose level (Figure 2B) (*F*_2,23_ = 15.185; *p* = 0.000). Immediately after AIS application, blood glucose level was significantly increased (Figure 2B) (*p* = 0.000), then dropped to basal level 30 min (Figure 2B) (*p* = 1.000) after the completion of AIS. We next investigated the PRDX6 expression level in the hippocampus after receiving 30 min of AIS. Western blot analysis revealed that mice that received AIS expressed significantly lower PRDX6 than the home-caged group in the hippocampus (Figure 2D) (*t*_6_ = 3.449, *p* = 0.014).

**Figure 3:**
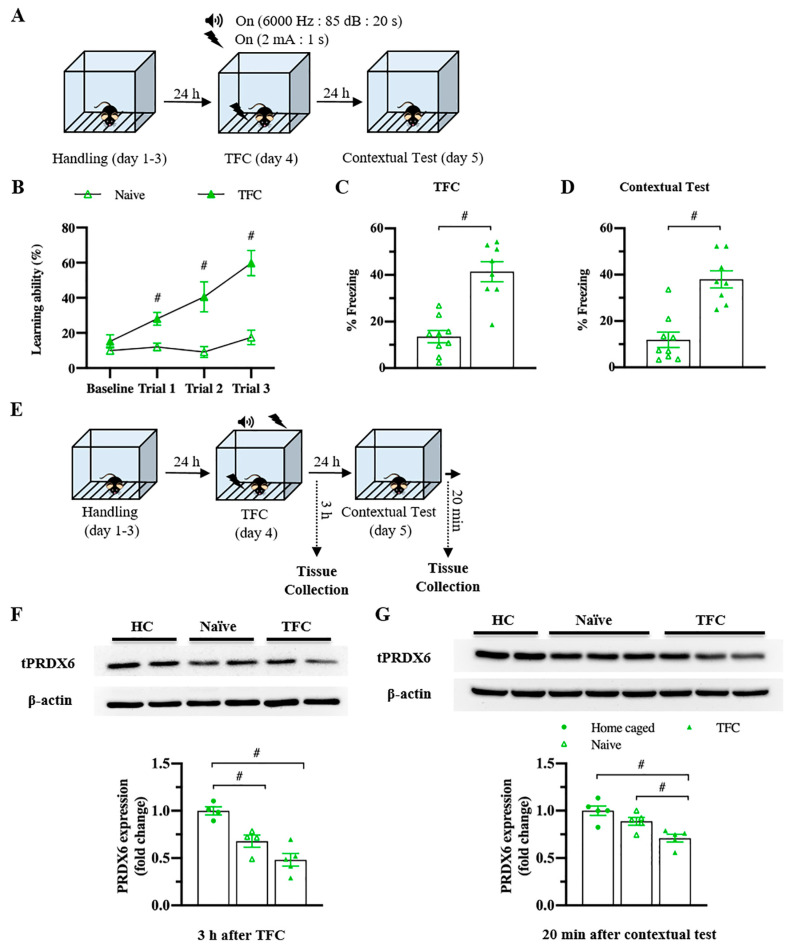
Trace fear conditioning (TFC) suppressed the expression level of PRDX6 in the hippocampus. (**A**) Tissue samples were collected and proteins were extracted from the hippocampus 3 h after the TFC and 20 min after a contextual test. (**B**) Freezing percentages of baseline and after each tone-shock pair presented as a learning curve (*n* = 8–9 mice per group, two-way repeated measure ANOVA for within group and Student’s *t*-test for between group). (**C**) Total percentage of freezing during the TFC. (**D**) Percentage of freezing during a contextual test. (**E**) The procedure for TFC and tissue samples collection. (F; upper panel) Immunoblotting of PRDX6 expression levels. (**F**; lower panel) Quantification data for PRDX6 expression levels 3 h after TFC (one-way analysis of variance (ANOVA) with LSD’s post hoc test). (**G**; upper panel) Immunoblotting of PRDX6 expression levels. (**G**; lower panel) Quantification data for the PRDX6 expression levels 20 min after a contextual test. All data represent the mean ± the SEM. # *p* < 0.05. PRDX6, peroxiredoxin 6; TFC, trace fear conditioning; HC, home caged.

To understand whether the levels of PRDX6 would be altered under a different type of stress, we investigated the hippocampal PRDX6 levels after TFC (Figure 3A). No difference in the percentage of baselines freezing was detected between naïve and trained mice (Figure 3B) (*t*_18_ = −0.375, *p* = 0.712). Trained mice showed significantly increased freezing percentages during three trials (Figure 3B) (trial 1: *F*_1,18_ = 14.342; *p* = 0.001; trial 2: *F*_1,18_ = 17.018; *p* = 0.001; trial 3: *F*_1,18_ = 28.781; *p* = 0.000). Total freezing behavior of the trained group was significantly higher than that of the naïve group (Figure 3C) (*t*_17_ = −6.411, *p* = 0.000), indicating they were able to learn the task. Trained mice also displayed higher freezing response to the conditioned context than the naïve mice (Figure 3D) (*t*_16_ = −3.563, *p* = 0.003). Three hours after TFC (Figure 3E), hippocampal PRDX6 expression level was decreased in the naïve and TFC groups compared with the home-caged group (Figure 3F) (*F*_2,12_ = 18.531; *p* = 0.000). Twenty minutes after the fear memory retrieval test for the conditioned context (Figure 3E), PRDX6 was decreased in the TFC group compared with naïve and home-caged groups (Figure 3G) (*F*_2,14_ = 10.858; *p* = 0.002). The results confirmed that TFC leads to a reduction in hippocampal PRDX6 as well as AIS.

### 3.3. Decreased H_2_O_2_ Level in the Hippocampal CA1, Basolateral Amygdala and Medial Prefrontal Cortex in Response to AIS in Prdx6^−/−^ Mice

**Figure 4:**
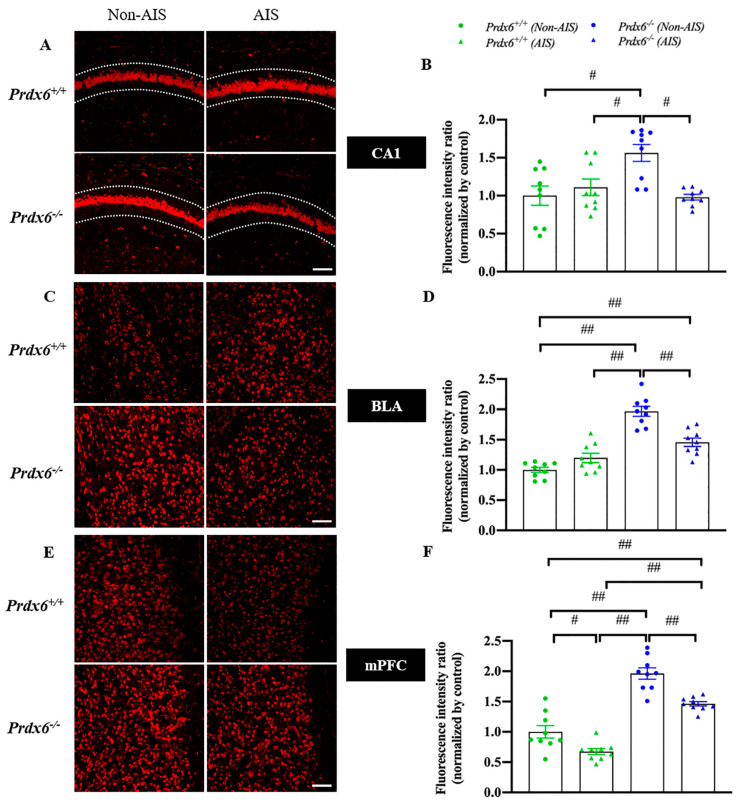
H_2_O_2_ levels were reduced in the hippocampal CA1, basolateral amygdala, and medial prefrontal cortex of *Prdx6^−/−^* mice with AIS. DHE staining and quantitative results of the (**A**,**B**) hippocampal CA1, (**C**,**D**) BLA, and (**E**,**F**) mPFC (*n* = 3 per group). All data represent the mean ± the SEM. # *p* < 0.05 and ## *p* < 0.001. AIS, acute immobilization stress; PRDX6, peroxiredoxin 6, BLA, basolateral amygdala, mPFC, medial prefrontal cortex. Bar = 100 μm.

To investigate whether PRDX6 affects H_2_O_2_ levels in mice after AIS, we measured the H_2_O_2_ levels by DHE staining in the hippocampal CA1, basolateral amygdala (BLA), and medial prefrontal cortex (mPFC) of *Prdx6^+/+^* and *Prdx6^−/−^* mice, with or without AIS. Our results showed that *Prdx6^−/−^* mice without receiving AIS had higher H_2_O_2_ levels in the hippocampal CA1 (Figure 4A,B, *F*_3,35_ = 7.162; *p* = 0.001), BLA (Figure 4C,D, *F*_3,35_ = 37.266; *p* = 0.001), and mPFC (Figure 4E,F, *F*_3,35_ = 54.506; *p* = 0.001) than *Prdx6^+/+^* mice. *Prdx6^+/+^* mice receiving AIS had significantly decreased H_2_O_2_ levels in mPFC compared with *Prdx6^+/+^* mice without AIS (Figure 4E,F). The H_2_O_2_ levels in *Prdx6^−/−^* mice with AIS were significantly reduced in hippocampal CA1 (Figure 4A,B), BLA (Figure 4C,D), and mPFC (Figure 4E,F) when compared with *Prdx6^−/−^* mice without AIS. In addition, *Prdx6^−/−^* mice with AIS demonstrated higher H_2_O_2_ levels in mPFC than *Prdx6^+/+^* mice receiving 30 min of AIS (Figure 4E,F). These results indicated that the 30 min of AIS reduced H_2_O_2_ levels in the three brain regions of *Prdx6^−/−^* mice.

### 3.4. Prdx6^−/−^ Mice Exhibited an Abnormal Locomotion and Anxiety Response after AIS

**Figure 5:**
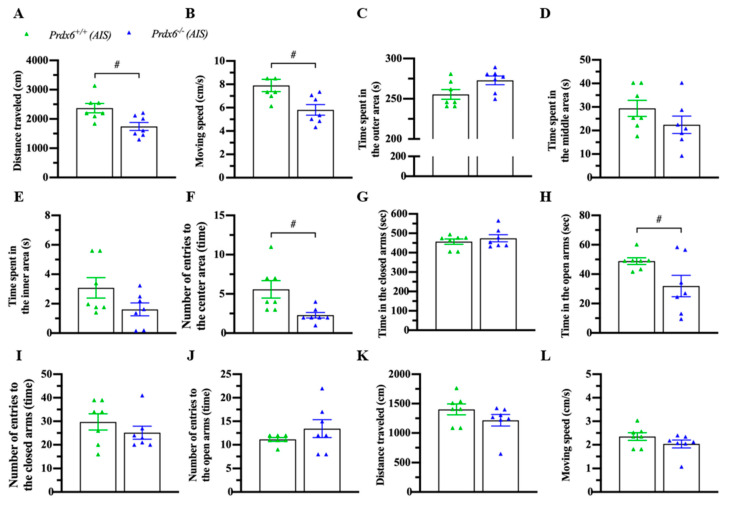
*Prdx6^−/−^* mice exhibited abnormal locomotor activity and anxiety response after AIS. Mice were immobilized for 30 min and recovered for another 30 min before the open field and elevated plus-maze tests (*n* = 7 per group). (**A**) The traveling distance and (**B**) moving speed of *Prdx6^−/−^* and *Prdx6^+^^/^^+^* mice for 10 min after AIS. The time spent in (**C**) the outer, (**D**) the middle, and (**E**) the inner areas of an open field chamber. The frequency of entries into (**F**) the center area of an open field chamber. Time spent in (**I**) closed arms and (**J**) open arms of an elevated plus-maze. The number of entries into the (**G**) open arms and (**H**) closed arms. (**K**) The traveling distance and (**L**) moving speed of both *Prdx6^−/−^* and *Prdx6^+^^/^^+^* mice in an elevated plus-maze after AIS. All data represent the mean ± the SEM. # *p* < 0.05. PRDX6, peroxiredoxin 6.

Acute immobilization can affect locomotion and anxiety response [34,35]. To confirm the phenomena, mice were immobilized for 30 min and kept in their home cage for another 30 min before a locomotion test with an open field and an anxiety behavior test with an elevated-plus maze. Interestingly, AIS caused less traveling distance (Figure 5A) (*t*_12_ = 2.983; *p* = 0.011) and lower moving speed (Figure 5B) (*t*_12_ = 2.985; *p* = 0.011) of *Prdx6^−/−^* mice. No difference was detected in time spent in outer (Figure 5C) (*t*_12_ = −2.146; *p* = 0.053), middle (Figure 5D) (*t*_12_ = 1.387; *p* = 0.191), and inner (Figure 5E) (*U* = 13; *p* = 0.141) areas between *Prdx6^−/−^* and *Prdx6^+/+^* mice. Moreover, *Prdx6^−/−^* mice entered the inner area less often than their wild-type littermates (Figure 5F) (*t*_12_ = 2.817; *p* = 0.016). In the elevated-plus maze test, there was no difference in time spent in closed arms (Figure 5G) (*U* = 21; *p* = 0.654). However, *Prdx6^−/−^* mice spent significantly less time in open arms compared with wild-type littermates (Figure 5H) (*t*_12_ = 2.213; *p* = 0.047) after receiving 30 min of AIS. No significant difference was recorded in entering frequency into closed (Figure 5I) (*U* = 19; *p* = 0.479) and open arms (Figure 5J) (*U* = 17; *p* = 0.324) of the elevated-plus maze between genotypes. The traveling distance (Figure 5K) (*U* = 14; *p* = 0.179) and moving speed (Figure 5L) (*U* = 14; *p* = 0.179) of *Prdx6^−/−^* mice on an elevated plus maze were comparable to those of wild-type mice.

### 3.5. The Prdx6^−/−^ Mice Demonstrated Lower Memory Retrieval to Context after AIS Compared with Non-AIS Prdx6^−/−^ Group

**Figure 6:**
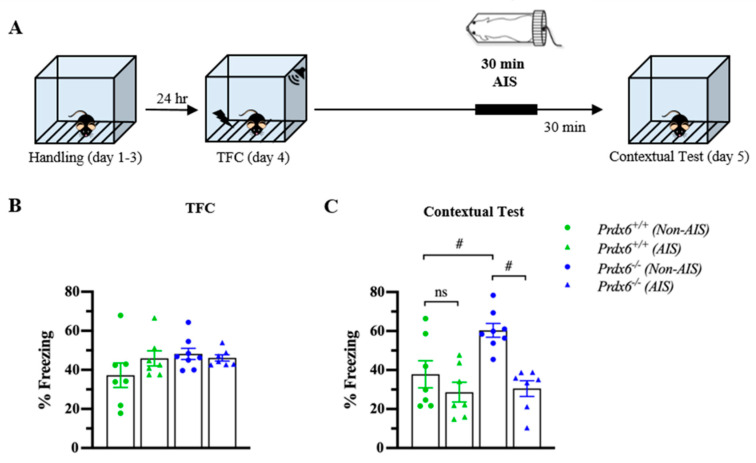
*Prdx6^−/−^* mice with AIS demonstrated significantly lower memory retrieval to context than those without AIS. (**A**) Schematic representation of the experimental procedure. After TFC, mice received 30 min of immobilization and recovered for another 30 min before the contextual test. (**B**) The percentage of total freezing of *Prdx6*^+/+^ or *Prdx6^−/−^* mice during the TFC (*n* = 7–8 per group, one-way analysis of variance (ANOVA) followed by Bonferroni’s post hoc test). (**C**) The percentage of freezing to context. All data represent the mean ± the SEM. # *p* < 0.05. PRDX6, ns; not significant, peroxiredoxin 6; AIS, acute immobilization stress; TFC, trace fear conditioning.

Post-traumatic stress disorder (PTSD) is a stress-related psychiatric disorder associated with the dysregulation of HPA axis activity [36]. We next used AIS to investigate whether the lack of PRDX6 affects memory performance under acute stress conditions. In this experiment, mice received 30 min of immobilization 1 h before the contextual test (Figure 6A). Total freezing percentage during TFC were similar among groups (Figure 6B) (*F*_3,28_ = 1.524; *p* = 0.233). The contextual test was performed 24 h after training. To further examine the effect of AIS on the ability to retrieve contextual fear memory, thirty minutes of AIS was applied to trace fear-conditioned mice 1 h before the beginning of the memory testing. We observed a significant difference in contextual fear memory retention (Figure 6C) (*F*_3,28_ = 9.295; *p* = 0.000). Freezing response to the context of the *Prdx6^−/−^* mice was higher than that of the wild-type mice for the non-AIS groups (Figure 6C) (*p* = 0.011). We detected that AIS reduced memory retrieval to the context in the *Prdx6^−/−^* mice compared with the non-AIS *Prdx6^−/−^* mice (Figure 6C) (*p* = 0.001), and the freezing percentage was similar to the wild-type level (Figure 6C) (*p* = 1.000, *Prdx6*^+/+^ with AIS vs. *Prdx6^−/−^* with AIS).

## 4. Discussion

The present study identifies the PRDX6 function in anxiety behavior and memory performance upon acute stress. We found that peroxiredoxin 6 (PRDX6) expression was downregulated in response to glucocorticoid (GC) treatment and upon acute immobilization stress (AIS). In addition, lack of the *Prdx6* gene leads to increased H_2_O_2_ levels in the hippocampus, amygdala, and medial prefrontal cortex, which can be reduced by AIS. We also demonstrated that *Prdx6^−/−^* mice exhibited anxiety behavior and attenuated contextual fear memory responding to AIS.

Both AIS and TFC can activate the hypothalamus–pituitary–adrenal (HPA) axis and the release of GC to the bloodstream to elicit emotional responses [37,38,39]. Excessive GC treatment has been found to activate ROS production in cells [40,41] and in the brains of animals and humans [42,43], while suppressing various antioxidant enzymes [43]. A decreased PRDX6 expression level within a short time may be involved in posttranslational modification of the protein. A previous study revealed that sumoylation of PRDX6 at its lysine 122 and 142 amplifies its enzymatic activity and stability [44], which may explain how PRDX6 level was decreased after GC treatment and contextual tests. Further investigations of how acute stress affects sumoylation and regulates expression levels of stress response proteins are needed. Several studies reported that PRDX6 expression level is regulated by nuclear factor erythroid 2-related factor 2 (NRF2) [45,46,47], in which its transactivation on targeting genes can be suppressed by activation of GC receptors [48]. The change of expression level of PRDX6 in vitro after GC treatment and in vivo after AIS and TFC may be through negative regulation of nuclear factor erythroid 2-related factor 2 (NRF2) by GC. Further study is necessary to verify whether NRF2 activity is related to GC and PRDX6 levels after AIS and TFC.

PRDX6 is expressed in the mice’s hippocampus, amygdala, and prefrontal cortex [21], suggesting its role in protecting oxidative stress occurred within these brain regions under stress conditions. Here, we detected elevated hydrogen peroxide (H_2_O_2_) levels in the hippocampus, amygdala, and medial prefrontal cortex of *Prdx6^−/−^* mice at basal condition, supporting the role of PRDX6 in oxidative defense mechanism [46,49,50]. We recorded that H_2_O_2_ levels after AIS were reduced in the hippocampus, amygdala, and medial prefrontal cortex of *Prdx6^−/−^* mice. It is known that optimal levels of GC can inhibit the production of ROS [41,51]. Therefore, the release of GC after experiencing 30 min of AIS may help lower H_2_O_2_ levels in the three brain regions of *Prdx6^−/−^* mice.

When exposed to stressful stimuli, fear and anxiety can become debilitating. Additionally, these maladaptive responses are related to the hippocampus, amygdala, and medial prefrontal cortex [52,53,54,55]. The brain consumes a large amount of oxygen and produces a high level of free radicals which make it sensitive to oxidative damage [56]. Increased ROS is correlated with brain disorders, including cognitive impairment and anxiety [57,58,59]. In adult rats, oxidative stress in the hippocampus and prefrontal cortex induces anxiety behavior and decreased locomotor activity [59,60]. Several antioxidant enzymes such as superoxide dismutase (SOD), catalase (CAT), and glutathione peroxidases (GPx) have been reported to be responsible for the elimination of excessive ROS [61]. Additionally, H_2_O_2_ can be reduced to water and oxygen either by catalase (CAT) or glutathione peroxidase (GPx) [62]. Since PRDX6 exerts GPx activity that can reduce H_2_O_2_ [63,64], it is not surprising that PRDX6 depletion gives rise to the excessive level of H_2_O_2_ in the hippocampus, basolateral amygdala and medial prefrontal cortex of *Prdx6^−/−^* mice. Subjecting the mice to 30 min of AIS is insufficient to reduce H_2_O_2_ level in the prefrontal cortex to wild-type level. A study in rats demonstrated that the prefrontal cortex is more sensitive to oxidative stress than the hippocampus following chronic isolation stress [65]. Thus, the AIS-induced anxiety response of *Prdx6^−/−^* mice may be due to H_2_O_2_ overload in the medial prefrontal cortex.

Contextual fear memory formation requires interactions between the hippocampus, amygdala, and prefrontal cortex [52]. During fear memory retrieval, the amygdala receives fear-related contextual information from the hippocampus [52]. This updated information is then relayed to the prefrontal cortex for further evolution before expressing a fear response. At the same time, the inhibitory signal sent from the ventral part of the prefrontal cortex to the central amygdala tightly regulates fear response. Functional change of either of these brain regions thus leads to dysregulation of fear memory retrieval [21]. We recently reported that the *Prdx6^−/−^* mice displayed excessive contextual fear memory accompanied by hyperphosphorylation of ERK1/2 in the hippocampus during the retrieval process [21]. The present study confirms that PRDX6 participates in the regulation of fear memory retrieval. Acute stress has either negative or positive effects on fear memory processes, depending on the intensity of the stressor and timing [2,66]. Both AIS and excessive ROS level can induce memory retrieval deficit [67,68,69]. The missing antioxidant effect of PRDX6 caused an imbalance of oxidant/antioxidant ratio may be responsible for attenuated fear memory retrieval to context after AIS.

## 5. Conclusions

The present study is the first to report the role of PRDX6 in mediating anxiety behavior and memory performance in response to AIS, and in controlling H_2_O_2_ levels in the brain. Furthermore, enhanced stress susceptibility of the *Prdx6^−/−^* mice suggests that PRDX6 can be a therapeutic target for treating stress-related disorders such as PTSD.

## Data Availability

The data that support the findings of this study are available from the corresponding author upon reasonable request.
